# The prescience of paleoclimatology and the future of the Antarctic ice sheet

**DOI:** 10.1038/s41467-018-05001-1

**Published:** 2018-07-16

**Authors:** Eric J. Steig, Peter D. Neff

**Affiliations:** Department of Earth and Space Sciences, University of Washington, Box 351310, Seattle, WA 98195 USA

## Abstract

The emerging view that the West Antarctic ice sheet is in the early stage of collapse owes as much to paleoclimatology as to contemporary observations.

The stability of the Antarctic ice sheet in the face of anthropogenic climate change is the central question in polar science. The retreat of glaciers and loss of ice shelves on the Antarctic Peninsula is well-documented^1^, but the processes are distinct from those affecting the much larger West and East Antarctic ice sheets. Our knowledge of the ice sheets has grown rapidly, thanks to advances in remote sensing and to dedicated field campaigns, which have provided an unprecedented view of the surface, interior, and bed, as well as the conditions in the surrounding oceans and atmosphere. It was discovered in the mid 1990s that there is rapid melting under the ice shelf of Pine Island Glacier^2^ one of the major outlet glaciers that drains West Antarctica into the sea. Shortly thereafter, observations showed that Pine Island Glacier and other outlet glaciers were retreating and thinning^3,4^. It is now clear that most of the margin of the West Antarctic ice sheet – and parts of East Antarctica as well – is undergoing rapid retreat^5^, and that the proximal cause is the erosion of ice shelves by warm ocean water^6^, amplified by intrinsic instabilities in glacier dynamics^7^.

Our contemporary understanding of the state of the West Antarctic ice sheet is often traced to the prescience of Mercer^8^, who 40 years ago suggested that anthropogenic climate warming would destabilize ice shelves, leading to ice sheet collapse. Recent observations certainly support Mercer’s understanding of the crucial role of ice shelves. Yet a solicitation of researchers’ views conducted as recently as 2013 concluded that expert opinion is both “uncertain and undecided” on whether recent trends in Antarctic ice sheet behavior are owing to natural variability, or are a response to climate change^9^. The reason for this ambiguity is simple: the record of direct observations of Antarctic ice sheet change is insufficient in length. The earliest application^10^ of satellite radar interferometry to obtain flow velocities for the Antarctic ice sheet was in 1993, and most other observational products, such as the satellite altimetry critical for evaluating thinning rates, are even more recent. Modern geophysical observations of the ice sheet thus comprise a period less than the standard textbook requirement for a representative climatology – 30 years – against which trends can be evaluated. This is surely too short a time to have observed the Antarctic ice sheet, characterized by intrinsic variability on even longer timescales than the atmosphere, and expect a definitive answer. Unless we are willing to wait another few decades or more – by which time the answer may well be clear, but no longer as useful – we need to incorporate information from paleoclimatology.

Mercer’s insight that the West Antarctic ice sheet is vulnerable was motivated by emerging paleoclimate evidence. Data from deep sea sediment cores^11^ had established that sea level was several meters higher than present during the last interglacial period (~132–116 thousand years ago), and it was soon recognized that the Antarctic ice sheet must have been considerably smaller^12^. Sediment cores drilled beneath the present-day ice sheet have since shown that the West Antarctic ice sheet collapsed at least once in the last million years^13^. Sea level during the last interglacial rose rapidly to between 5 and 9 m higher than present, a finding that is difficult to explain without a major contribution from Antarctica^14^. Such evidence remains the strongest indication of ice sheet sensitivity to climate change.

Modern numerical ice-sheet modeling is also informed by paleoclimate evidence. Simulations of the future behavior of the ice sheet rely on estimates of past ice sheet configuration and past climate forcing (i.e., temperature and snow accumulation rate) for initialization and validation. Difficulties in matching the output of model simulations to geological constraints on paleo-ice sheet configuration and sea level have demanded the implementation of new model physics: a more-efficient mechanism for ice-shelf calving than had been conventionally assumed^15^. Simulations with this model physics cause West Antarctic ice sheet collapse during the last interglacial, provided that strong warming of the Southern Ocean is assumed as a boundary condition^16^. Evidence for such warming can indeed be found in the paleoclimate record^17^. The same model physics, under projected anthropogenic climate forcing, leads to rapid deglaciation of West Antarctica^16^. Thus, evidence of the past response of the ice sheet to paleoclimate informs our understanding of its future.

Paleoclimate data also provide context for the ice sheet changes being observed today (Fig. 1). Increased delivery of warm ocean water to the margin of the ice sheet, implicated in the current phase of retreat, is the consequence of changing ocean circulation, driven by changes in the atmosphere. Investigation of this atmosphere–ocean forcing relies heavily on climate reanalysis data, which are reliable in the Antarctic region only since 1979; there is only one weather station in West Antarctica (Byrd Station) that provides continuous meteorological observations further back in time, and that begins only in the 1957 International Geophysical Year. Ice cores, on the other hand, provide information stretching back thousands of years. Annually-resolved ice cores show that the major El Niño event of 1939–1942 had a significant impact on West Antarctic climate^19^ leading to the suggestion that this event was an important forcing of glacier retreat in the Amundsen Sea^20^. Sediment cores^21^ collected from beneath the Pine Island Glacier ice shelf have since shown that flooding of the continental shelf by warm Circumpolar Deep Water began around the 1940s. Data like these will be critical for determining when – and if – climate changes in Antarctica reach the point where, as Mercer put it, “a dangerous trend is under way.” To date, a definitive answer is available only for the Antarctic Peninsula, but the result from an ice core there is dramatic: summer melt now exceeds anything in the past millennium^22^.

**Fig. 1:**
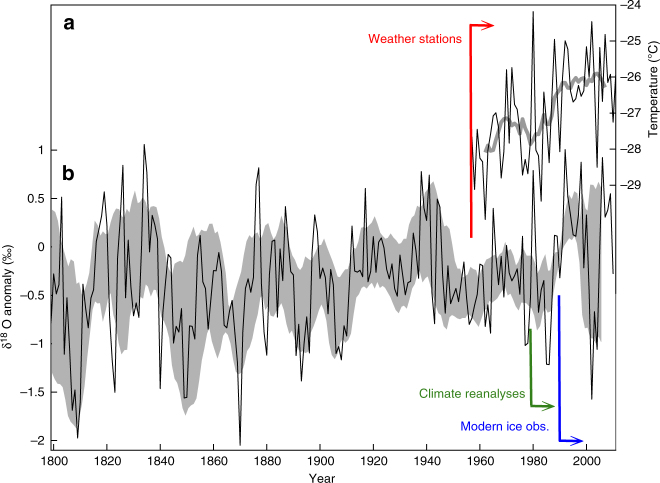
West Antarctic climate variability over the last 200 years. **a** Mean-annual temperature data at the Byrd weather station. **b** Oxygen isotope (δ^18^O) data from a collection of ice cores from the West Antarctic ice sheet. Gray shading shows decadal average with uncertainties. Vertical lines show the earliest year for which surface climate observations (red, 1957), climate reanalysis products (green, 1979) and spatially-complete geophysical ice-sheet observations (blue, ~1990) are available. Figure modified from Steig et al.^18^

Paleoclimate data, of course, have limitations compared with instrumental climate observations and modern geophysical imaging of ice sheet structure. Yet, the spatial coverage of ice cores is far greater than that of weather stations, and there is potential to use such records even more effectively, through the blending of proxy data with information from climate models to produce longer-term climate reanalysis products^23^. For such techniques to yield reliable information about the conditions most relevant to the ice dynamics, additional records will be needed from regions closer to the center of action. Cores from the numerous ice domes along the Amundsen and Bellingshausen Sea coasts of West Antarctica would be particularly valuable in this respect, though the logistical difficulties of working in this remote region have thus far prevented initiatives to obtain such cores from moving forward.

Somewhat remarkably, it still remains to be resolved whether the West Antarctic ice sheet collapsed during the last interglacial period. This uncertainty presents an important challenge that can only be answered definitively with paleoclimate and paleo-glaciological data. One approach is to obtain spatial reconstructions of climate using deep ice core records. A significantly smaller ice sheet would be associated with changes in weather patterns which should be detectable in ice cores from strategically-located sites such as Hercules Dome, inland of the Transantarctic Mountains in East Antarctica, adjacent to the West Antarctic ice sheet. Climate model results suggest that the climate at Hercules Dome would be especially sensitive to atmospheric circulation changes associated with ice-sheet collapse^24^. Another promising idea is to use borehole drilling to obtain samples of bedrock from beneath the current ice sheet, and then use cosmogenic nuclide measurements to determine bedrock exposure history, yielding information about times when the ice sheet was smaller^25^.

Some researchers have concluded that the West Antarctic ice sheet is already in the early stages of an irreversible collapse^26,27^. Certainly, the current retreat of the margins of the Antarctic ice sheet is remarkable, and expert opinion on whether such trends are worrisome has probably moved somewhat from “uncertain” towards “likely” in the last few years. Observations from paleoclimate have played a powerful role in this emerging view, by providing critical boundary conditions for ice sheet models and elucidating the conditions under which ice sheet collapse may have occurred in the past. Paleoclimate data also provide an important role in balancing the interpretation of modern observations against the longer-term context in which ongoing ice sheet changes are occurring. Such complementary information will continue to be necessary as the ice sheet continues to change in a warming world.
